# PyCaret for Predicting Type 2 Diabetes: A Phenotype- and Gender-Based Approach with the “Nurses’ Health Study” and the “Health Professionals’ Follow-Up Study” Datasets

**DOI:** 10.3390/jpm14080804

**Published:** 2024-07-29

**Authors:** Sebnem Gul, Kubilay Ayturan, Fırat Hardalaç

**Affiliations:** Department of Electrical and Electronics Engineering, Faculty of Engineering, Graduate School of Natural and Applied Sciences, Gazi University, Ankara 06570, Turkey; sebnem.gul@gazi.edu.tr (S.G.); kubilay.ayturan@gazi.edu.tr (K.A.)

**Keywords:** type 2 diabetes mellitus, PyCaret, machine learning, prediction, feature importance plot, SHAP value

## Abstract

Predicting type 2 diabetes mellitus (T2DM) by using phenotypic data with machine learning (ML) techniques has received significant attention in recent years. PyCaret, a low-code automated ML tool that enables the simultaneous application of 16 different algorithms, was used to predict T2DM by using phenotypic variables from the “Nurses’ Health Study” and “Health Professionals’ Follow-up Study” datasets. Ridge Classifier, Linear Discriminant Analysis, and Logistic Regression (LR) were the best-performing models for the male-only data subset. For the female-only data subset, LR, Gradient Boosting Classifier, and CatBoost Classifier were the strongest models. The AUC, accuracy, and precision were approximately 0.77, 0.70, and 0.70 for males and 0.79, 0.70, and 0.71 for females, respectively. The feature importance plot showed that family history of diabetes (famdb), never having smoked, and high blood pressure (hbp) were the most influential features in females, while famdb, hbp, and currently being a smoker were the major variables in males. In conclusion, PyCaret was used successfully for the prediction of T2DM by simplifying complex ML tasks. Gender differences are important to consider for T2DM prediction. Despite this comprehensive ML tool, phenotypic variables alone may not be sufficient for early T2DM prediction; genotypic variables could also be used in combination for future studies.

## 1. Introduction

Type 2 diabetes mellitus (T2DM) is a chronic metabolic disorder that affects millions of people worldwide, posing significant health and economic burdens. Early detection and prevention of T2DM are crucial to reducing its complications and improving the quality of life of patients [1]. Predicting diabetes allows for earlier detection and intervention, potentially delaying or preventing disease progression. This aligns with personalized medicine’s emphasis on proactive healthcare. However, the current diagnostic methods for T2DM, such as the oral glucose tolerance test (OGTT) and the glycated hemoglobin (A1C) test, can be invasive, costly, and time-consuming.

Phenotypic data can provide valuable insights into the risk factors and pathophysiology of T2DM. Phenotypic data include anthropometric measurements, biochemical markers, lifestyle habits, medical history, and family history. Machine learning (ML) techniques, which are computational methods that learn from data and make predictions, can leverage phenotypic data to build predictive models for T2DM [2]. ML techniques have several advantages over conventional statistical methods, such as the ability to handle high-dimensional and nonlinear data, discover complex patterns and interactions, and improve accuracy and generalization [3,4,5].

Several studies have applied different ML techniques to predict T2DM by using phenotypic data from various populations. For example, Yu et al. (2010) used support vector machine (SVM) to classify instances of diabetes by using data from the National Health and Nutrition Examination Survey (NHANES) [6]. Deberneh and Kim utilized five ML algorithms for the prediction of T2DM by using both laboratory results and phenotypic variables [2]. Anderson et al. used a reverse engineering and forward simulation (REFS) analytical platform that relies on a Bayesian scoring algorithm to create prediction-model ensembles for progression to prediabetes or T2DM in a large population [7]. Cahn et al. used electronic medical record (EMR) data from The Health Improvement Network (THIN) database, which represents the UK population, to identify prediabetic individuals [8]. Shin et al. used Logistic Regression (LR), Decision Tree, Random Forest (RF), eXtreme Gradient Boosting (XGBoost), Cox regression, and XGBoost Survival Embedding (XGBSE) for the prediction of diabetes [9]. Gul et al. investigated the prediction value of phenotypic variables (body mass index, cholesterol, familial diabetes history, and high blood pressure) with LR [10]. Dinh et al. achieved high area under curve (AUC) scores with and without laboratory data with LR, SVM, and three ensemble models (RF, Gradient Boosting-XGBoost, and a weighted ensemble model) [11]. Viloria et al. used SVM to predict T2DM by only using body mass index (BMI) and blood glucose concentration [12]. Wang et al. compared XGBoost, SVM, RF, and K-Nearest Neighbor (K-NN) algorithms to predict the risk of T2DM [13].

Previous studies have shown that ML techniques can be used to predict T2DM by using phenotypic data. However, these studies have several limitations. First, they focus on a few ML algorithms or compare them in isolation without considering the full range of existing methods or novel algorithms. Second, some of these studies use laboratory results that are directly related to glucose metabolism and may not reflect other aspects of phenotypic variation. Another limitation of these studies is their lack of consideration for gender disparities in risk factors, disease outcomes, and model performance for T2DM. Additionally, some studies relied on datasets that were either small or of questionable quality [13]. Despite these limitations, the previous studies provide promising evidence that ML techniques can be used to develop accurate and robust models for predicting T2DM risk.

This study aimed to address the limitations of previous studies by using PyCaret, an open-source, low-code ML library in Python that automates ML workflows [14]. PyCaret allows for the simultaneous evaluation of multiple ML algorithms, including newly developed ones, such as XGBoost, LightGBM, and CatBoost, for predicting T2DM. PyCaret eliminates the need for extensive domain expertise, reduces the analysis time, and allows one to obtain more comprehensive evaluation metrics.

Another aim of this research was to explore the differences between male and female populations for predicting T2DM. By analyzing how phenotype and gender interact, we can identify risk factors that are more prominent in one sex compared with the other. This knowledge can allow healthcare providers to tailor personalized screening and prevention strategies for men and women.

## 2. Materials and Methods

### 2.1. Dataset Overview

Raw data from controls and patients were obtained from the “Nurses’ Health Study” (NHS), an all-female cohort, and the “Health Professionals’ Follow-up Study” (HPFS), an all-male cohort. Data are available at the database of Genotypes and Phenotypes (dbGaP) under accession phs000091.v2.p1 and were obtained with permission [15].

The NHS and HPFS are well-established cohorts that are part of the Genes and Environment Initiatives (GENEVA). In addition to investigating the genetic factors contributing to the development of T2DM, they also aim to explore the role of environmental exposure. These cohorts offer a resource for studying the genetic and environmental factors associated with T2DM. Participants in both cohorts completed comprehensive mailed questionnaires regarding their medical history and lifestyle.

The “Nurses’ Health Study” (NHS), initiated in 1976 with 121,700 female nurses aged 30–55 years, and the “Health Professionals’ Follow-up Study” (HPFS), established in 1986 with 51,529 male US health professionals aged 40–75 years, both began by collecting data on medical history and lifestyle through mailed questionnaires [16]. Every two years since, participants in both cohorts have completed self-administered questionnaires to update information on medical history, lifestyle factors (diet, exercise, and smoking), demographics (age and ethnicity), and family history of diabetes. This study leverages data from the “Nurses’ Health Study” and the “Health Professionals’ Follow-up Study” as part of the Gene Environment Association Studies (GENEVA) initiative. GENEVA aims to identify novel genetic contributors to T2DM through a genome-wide association analysis. Blood samples were collected for genotyping in 1989–1990 for the NHS and in 1993–1995 for the HPFS. While both studies initially enrolled a large number of participants, a subset of approximately 6000 individuals with T2DM (cases) and healthy controls was selected for this specific project for GENEVA [15]. Our analysis focuses on the phenotypic data collected from this selected group.

### 2.2. Variables

The variables used for analysis in this study are given in Table 1.

Categorical variables were used as they are, with no numeric conversion applied for analysis.

### 2.3. Data Preprocessing

The data contain information about the disease status of a total of 6033 individuals: 3429 females (NHS) and 2604 males (HPFS). The analysis focused on white individuals. Participants of other races (158), Hispanic (37), those with other types of diabetes (133), individuals without genotype ID (25), and first-degree relatives (8) of the participants were excluded. The characteristics of the remaining 5672 individuals, which included both controls and T2DM individuals, are presented in Table 2.

### 2.4. PyCaret Analysis

PyCaret is a low-code framework that enables the simultaneous execution of machine learning algorithms. When the main code is run, many embedded codes execute in a specified order, automating the machine learning analysis process. Instead of calculating accuracy in one code and precision in another, all codes are embedded and executed sequentially, creating comparison tables and graphics automatically in a short time. This enables researchers who may not have software expertise to perform machine learning analysis with minimal coding, provided they prepare the dataset properly.

PyCaret was used to analyze a dataset of 5672 individuals with 14 phenotypic variables for the male and female datasets and 15 variables, including gender, in the total dataset. The dataset was divided into male and female subsets, and the performance and features of 16 machine learning (ML) classification algorithms were compared for each subset.

PyCaret analysis was performed in the following order: import the necessary libraries and the data (as a .csv file), then preprocess the data by using PyCaret, display data features (numeric or categorical), and handle missing data. Since the rows with missing values accounted for 3.5% of the dataset, with a maximum rate of 1.5% in individual features, no data points were dropped. PyCaret used a simple imputation method to address the missing data, utilizing the mean for the imputation of numeric variables and the mode for the imputation of categorical variables.

**Figure 1:**
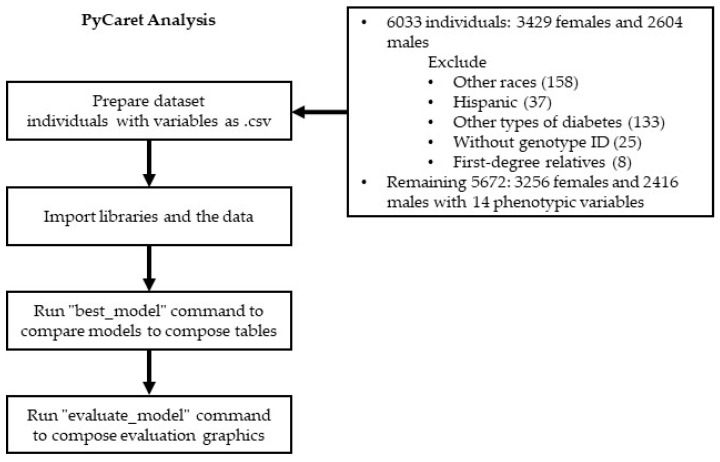
Process flow diagram.

Next, the classification command was used to compare the available classification algorithms. Once the results were available, the best algorithm was selected and performed 10-fold cross-validation for tuning the model’s hyperparameters. The model’s performance and robustness were evaluated by using stratified 10-fold cross-validation in a train–test split ratio of 70:30. The process flow diagram is presented in Figure 1. The analysis was implemented by using the Anaconda Navigator IDE with Python in the Jupyter Notebook (version 6.5.4) editor, along with PyCaret (version 3.2.0), on a 64-bit Windows 11 computer.

The analysis provided the following evaluation metrics: accuracy, area under curve (AUC), recall, precision, F1-score, kappa score, Matthew’s Correlation Coefficient (MCC), and analysis time (TT). A variable importance graph was also produced. A SHAP value graph was generated by using the “interpret_model” command.

The explanation of the performance metrics is given below:

- **Accuracy** is the percentage of correct predictions out of all predictions.
- **Precision** is the percentage of correct positive predictions out of all positive predictions.
- **Recall** is the percentage of correct positive predictions out of all actual positives.
- **F1-score** is a harmonic mean of precision and recall that balances both metrics.
- **Area under the curve** is a measure of how well a model can rank positive and negative examples correctly.
- **Kappa score** is a measure of agreement between the model’s predictions and the actual labels.
- **Matthew’s Correlation Coefficient** is used as a measure of the quality of binary classifications.

The kappa score and MCC are valuable metrics for evaluating the performance of a classifier in identifying true positives and negatives. This information can aid clinical decision making by minimizing the risk of misclassifying patients.

The formulas for these metrics are

$$Accuracy=\frac{\left(TP+TN\right)}{\left(TP+TN+FP+FN\right)}$$

$$Precision=\frac{TP}{\left(TP+FP\right)}$$

$$Recall=\frac{TP}{\left(TP+FN\right)}$$

$$F1-score=2\times \frac{\left(Precision\times Recall\right)}{\left(Precision+Recall\right)}$$

$$Kappa\;score=\frac{2\times (TP\times TN-FN\times FP)}{\left(TP+FP\right)\times \left(FP+TN\right)+\left(TP+FN\right)\times \left(FN+TN\right)}$$

$$MCC=\frac{\left(TP\times TN-FP\times FN\right)}{\sqrt{\left(TP+FP\right)\times \left(TP+FN\right)\times \left(TN+FP\right)\times \left(TN+FN\right)}}$$

TP: true positive
FP: false positive
TN: true Negative
FN: false negative

### 2.5. Statistical Analysis

SPSS software (version 29.0.0.0; SPSS Inc., Chicago, IL, USA) was used to analyze the numerical data. The Kolmogorov–Smirnov test was used to evaluate normality, and either the Student’s *t*-test or the Mann–Whitney U test was used for the statistical analysis of numeric variables, as appropriate. A chi-square test was used to examine the association between categorical variables by using Chi-Square Test Calculator [17]. Numerical variables were presented as means ± standard deviation (SD) and categorical variables as frequencies and percentages. The significance level was set at *p* < 0.05.

## 3. Results

### 3.1. The Results of the Statistical Analysis

The ages of the control and diabetic groups were similar. Statistical comparisons of the other numerical variables are provided in Table 2.

Overall, the table suggests that people with T2DM are more likely to have a higher body mass index, lower activity level, lower alcohol intake, lower cereal fiber intake, lower magnesium, and higher heme intake. It is noteworthy that the *p*-value for body mass index in females is much lower than in males.

Statistical comparisons of the categorical variables are provided in Table 3.

The findings suggest that certain factors may be associated with T2DM risk, such as having a family history of DM, high blood pressure, high cholesterol, and smoking status. It is important to note that this is an observational study, so it cannot establish causality. More research is needed to determine the causal relationships between these factors and T2DM.

### 3.2. The Results of the Machine Learning Analysis

#### 3.2.1. Learning Curve Analysis

**Figure 2:**
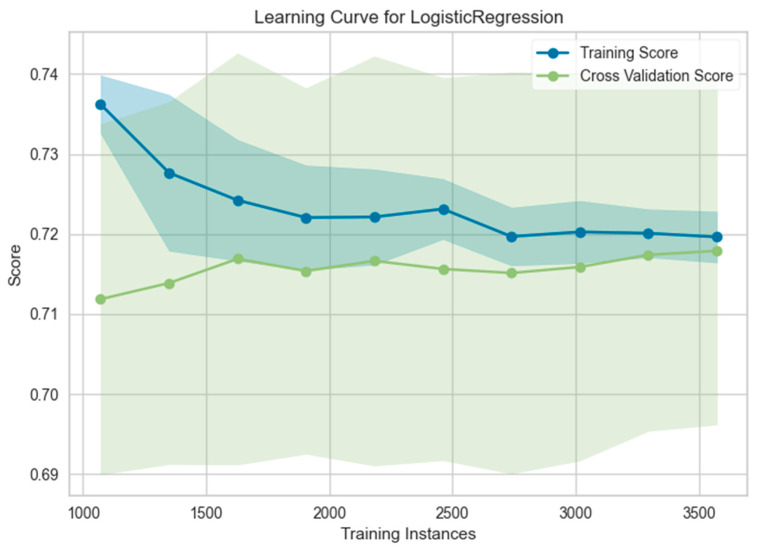
Learning curve for total (male + female) dataset.

A plot of training loss and validation loss against the number of training iterations provides a visual representation of the learning curve, which shows how error changes as the training set size increases. A learning curve is important for understanding whether a model is a good fit, overfit, or underfit. Figure 2 illustrates the learning curves for the Logistic Regression classifier trained on the combined male and female data. Initially, the validation score starts slightly higher than the cross-validation score. As the model encounters a wider variety of data points during training, the validation score gradually decreases and approaches the cross-validation score. This convergence indicates that the model is learning the underlying trends in the data effectively and can generalize this learning to new data, suggesting a good fit for the model. In simpler terms, the model is performing consistently on both the training data it is familiar with and the unseen data it will be used on in real-world applications. The plateauing of the learning curve in the middle suggests that the model’s performance has reached a point of diminishing returns for additional training with this specific dataset.

#### 3.2.2. Comparison of Algorithms for Prediction of T2DM

The performance of 16 different machine learning models on a classification task for predicting diabetes in the male-only, female-only, and total (male + female) data subsets is shown in Table 4, Table 5 and Table 6, respectively. The metrics used to evaluate the models were accuracy, AUC, recall, precision, F1-score, kappa score, and MCC. The highest score for each metric and subset is highlighted in yellow.

Ridge Classifier, Linear Discriminant Analysis (LDA), and LR were the top-performing models for the male-only data subset (Table 4), with similar scores on metrics. However, Ridge Classifier could not distinguish between the positive and negative classes, as indicated by its zero AUC value. LDA and LR had higher AUC values, around 0.77, than the other models.

For the female-only data subset (Table 5), LR, Ridge Classifier, and LDA achieved the best results on the analysis metrics. LDA had the highest AUC value, around 0.79, followed by LR with a similar AUC value. Ada Boost and CatBoost Classifiers had marginally lower AUC values, around 0.77 each.

For the total data subset (Table 6), LR, Gradient Boosting Classifier (GBC), and CatBoost Classifier were the best models on the analysis metrics. LR had the highest AUC value, around 0.79, followed by GBC and CatBoost Classifier with a moderate AUC value of 0.78.

#### 3.2.3. The Results of the Feature Importance Plot

**Figure 3:**
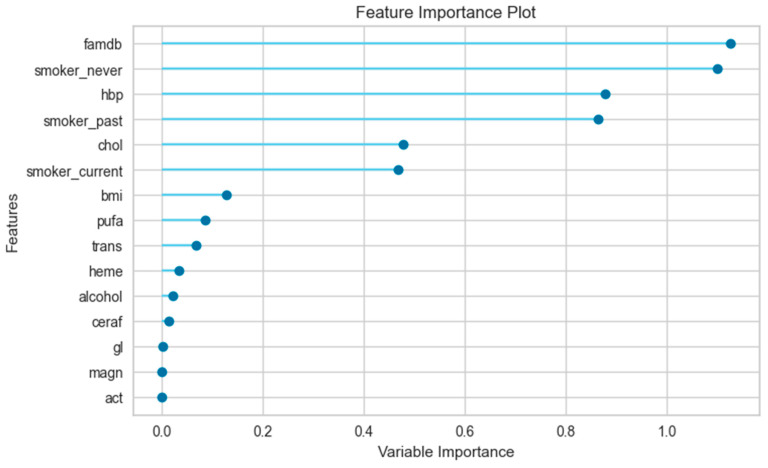
Feature importance plot for female-only data subset.

The feature importance plot in Figure 3 for the female-only data subset shows that the most informative feature for the prediction model was “famdb”, which has a variable importance of approximately 1.15. This means that this feature has a strong influence on the model’s performance and that removing or shuffling it would significantly reduce the accuracy or increase the error of the model. The other features, such as “smoker_never”, “hbp”, “smoker_past”, and “chol”, are the next most important features, with values between 0.4 and 1.1. It is important to note that this is just one interpretation of the variable importance plot.

**Figure 4:**
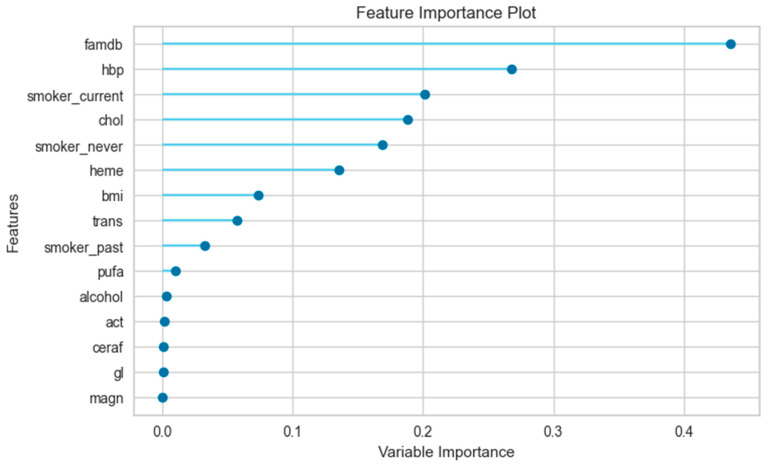
Feature importance plot for male-only data subset.

Figure 4 shows the feature importance plot for the male-only data subset. The most important feature for the prediction model is famdb, which has a variable importance of approximately 0.45. The other features that follow in terms of importance are hbp, smoker_current, chol, smoker_never, heme, and bmi. It can be noted that the value of the variables for males in the feature importance plot is lower than that for females.

**Figure 5:**
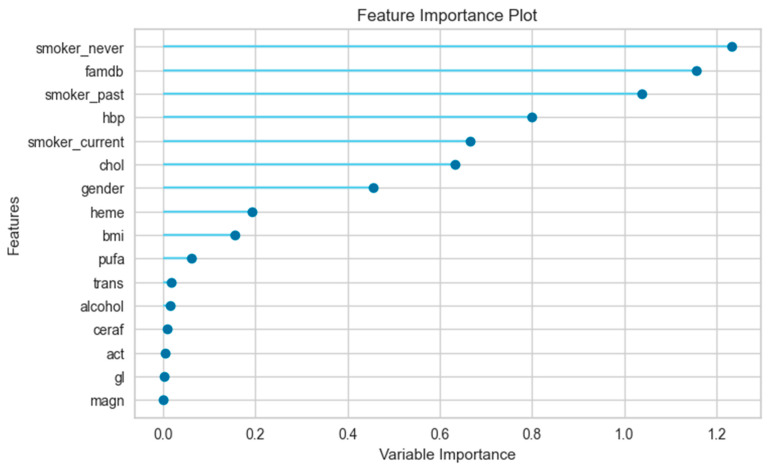
Feature importance plot for total (female + male) dataset.

Figure 5 shows that the most important feature for the prediction model is “smoker_never”, which has a variable importance of approximately 1.25 in total (female + male). The other features have variable importance values between 0.0 and 1.15, indicating that they have varying degrees of influence on the model’s performance. Some features, such as “famdb”, “smoker_past”, “hbp”, “smoker_current”, “chol”, and “gender”, have relatively high variable importance values, suggesting that they are important for the model. Other features, such as “bmi”, “pufa”, “trans”, and “alcohol”, have relatively low variable importance values, suggesting that they are less important for the prediction model.

By comparing the three figures, it is possible to observe how the importance of each feature varies across different data subsets. It can be noted that “gender” is one of the important factors for predicting diabetes in the total dataset, suggesting that gender is an important feature for modeling.

#### 3.2.4. SHAP Analysis

**Figure 6:**
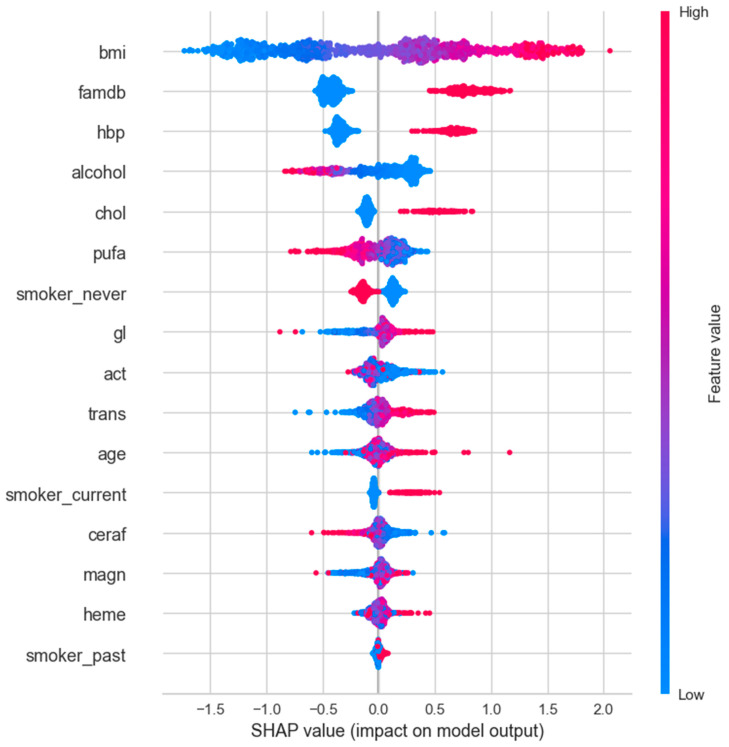
SHAP values for female-only data subset.

SHAP values correlation analysis was performed for variables with T2DM by using PyCaret analysis. Since the SHAP value function is only compatible with tree-based models for binary classification, the best-performing one was chosen among Decision Tree (dt), CatBoost, XGBoost, Extra Trees (et), LightGBM, and Random Forest (rf) to plot the SHAP value graph. Therefore, the CatBoost algorithm, which had the highest score among the tree-based models, was used for SHAP analysis. The results of the SHAP analysis are shown in Figure 4, Figure 5 and Figure 6 for the female-only, male-only, and total (female + male) datasets, respectively.

SHAP is a theoretical approach to explaining the model output of a machine learning model in a relatively easy way. SHAP values show the distribution of impacts that each feature has on the model output. They show which features are most crucial for the model’s performance. Unlike ROC and AUC analyses, SHAP values do not show model performance but rather interpret the impact of having a certain value for a given feature compared with the prediction we would make if that feature took some baseline value [18]. The SHAP figure aligns well and complements the statistical results by visually highlighting the behavior of variables in predicting diabetes.

The *y*-axis shows the features of the model, and the *x*-axis is the SHAP value of the associated features. Lower values of the feature are placed on the left, and higher values of the feature are placed on the right of the *x*-axis in the SHAP figures. The higher the SHAP value on the *x*-axis, the greater the impact on the model’s output. The red color indicates feature values that increase the likelihood of diabetes, while the blue color represents values that decrease the likelihood, whether on the left or right of the *x*-axis. The pink section, a combination of blue and red feature values mainly placed at or near the center of the *x*-axis, indicates that there is no clear relationship between feature values and SHAP values.

In females, while all higher values of features were associated with diabetes, lower values of alcohol, never having smoked, polyunsaturated fat, and cereal fiber intake favored diabetes. Activity, heme iron, and magnesium intake seemed to have little or no effect on diabetes in the current dataset (Figure 6).

**Figure 7:**
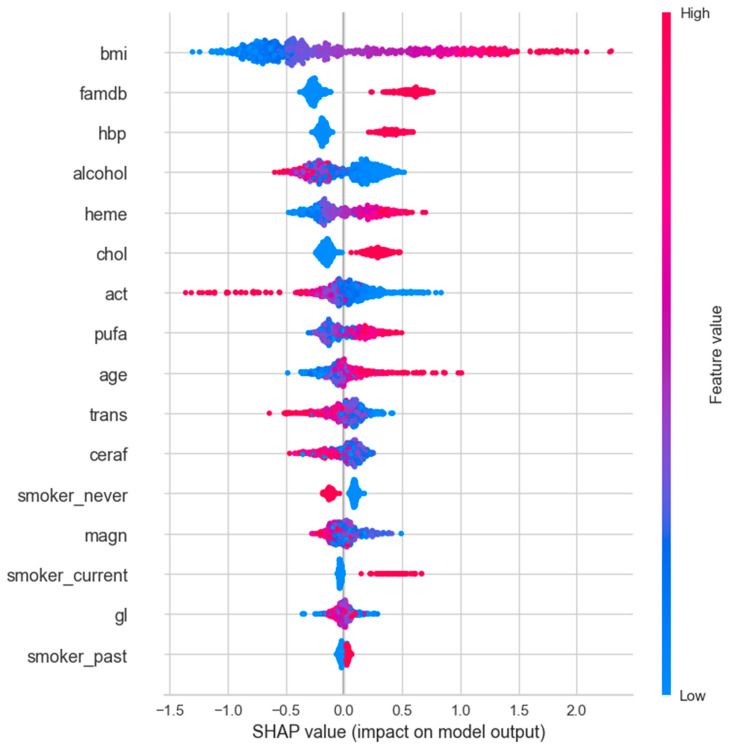
SHAP values for male-only data subset.

Similarly, in males, higher values of most features favored diabetes, while lower values of alcohol, activity, never having smoked, trans fat, and cereal fiber intake were in favor of diabetes. Glycemic load and magnesium intake appeared to have little or no effect on diabetes in this dataset (Figure 7).

**Figure 8:**
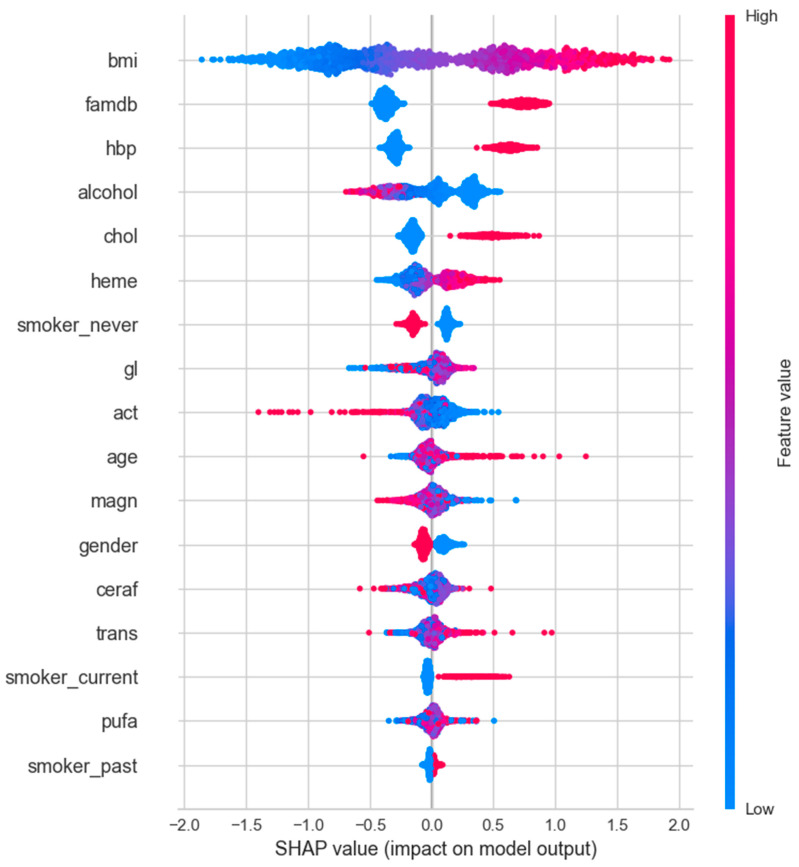
SHAP values for total (female + male) dataset.

When combining the male and female data, the effects of cereal fiber and polyunsaturated fat intake fade away in prediction. Lower levels of magnesium and higher trans fat intake were associated with diabetes. This suggests that combining male and female data can mask the true effect of some features on the prediction of diabetes (Figure 8).

## 4. Discussion

The use of artificial intelligence in healthcare has emerged in recent years, alongside the automation of ML, to improve the quality, efficiency, and effectiveness of healthcare [19]. ML algorithms, which are a set of rules that do not require explicit programming, allow computers to learn and make predictions from data. They are used for a variety of applications, such as natural language processing, image recognition, fraud detection, and disease prediction [20]. In the past, the use of the most prevalent ML algorithms simultaneously was infeasible due to the need for programming expertise and time-consuming testing. However, developments in information technologies have made it possible to run ML algorithms without knowing much code and to analyze many ML algorithms simultaneously.

Machine learning and statistical analysis were applied in the current study to investigate the factors associated with T2DM in the NHS and HPFS datasets. PyCaret classification analysis was used as a machine learning tool to run 16 classification algorithms simultaneously with minimal coding to predict T2DM. The performance of the models was evaluated by using various metrics, such as accuracy, AUC, recall, precision, F1-score, kappa, MCC, and TT (Sec). Feature importance plots and SHAP values were used to interpret the models and identify the most relevant features for prediction.

Ridge Classifier, LDA, and LR exhibited the best performance among models for the male-only data subset, all achieving similar scores. In contrast, for the female-only data subset LR, Ridge, and LDA were the top-performing models, also with similar scores. In the total data subset, LR, GBC, and CatBoost Classifier emerged as the best-performing models, demonstrating comparable scores.

The feature importance plot, one of the most commonly used explanation tools in machine learning analysis, was also utilized [21]. This tool shows how much each feature contributes to the prediction model, based on the change in accuracy or error when the feature is removed or shuffled [22]. The higher the variable importance, the more important the feature is for the model. However, this tool does not imply any causal relationship between the features and the outcome, as there may be other factors or interactions that affect the model. A feature may have high statistical significance but low variable importance if it does not improve the prediction model much.

The feature importance plot aids in understanding relevant features for the prediction model and identifying irrelevant or redundant ones. Additionally, it assists in selecting or eliminating features to enhance or simplify the model. However, it should be noted that the feature importance plot may vary depending on the type of machine learning technique, the dataset, and the evaluation metric used for measuring the model’s performance. Therefore, it should be interpreted with caution and in conjunction with other methods of machine learning analysis. The feature importance plot showed that features had different importance values for the prediction model in different data subsets. The most important features were “famdb”, “smoker_never”, and “hbp” in the female-only data subset, and “famdb”, “hbp”, and “smoker_current” in the male-only data subset. Furthermore, the order of the variables and their values differ across genders.

Dinh et al. used 123 variables for 1999–2014 and 168 variables for 2003–2014, including survey questionnaire and laboratory results [11]. They found that the AUCs were 73.7% and 84.4% without and with the laboratory data for prediabetic individuals, respectively. In another study, Lai et al. found that the GBM and Logistic Regression models performed better than the Random Forest and Decision Tree models. However, they used several laboratory data, such as fasting blood glucose, in their models, as well as BMI, high-density lipoprotein (HDL), and triglycerides, as the most important predictors [23]. The well-known Framingham Diabetes Risk Scoring Model (FDRSM) is a simple clinical model that uses eight factors, i.e., gender, age, fasting blood glucose, blood pressure, triglycerides, HDL, BMI, and parental history of diabetes, in order to predict the 8-year risk for developing diabetes by Logistic Regression models [24,25]. They also used 2 h post-OGTT in complex clinical models [24]. While the AUC was 0.72 in the simple model, it increased to 0.85 in the complex clinical model. The use of either blood glucose levels or OGTT in model creation clearly has a significant impact on model performance alone. However, it is crucial to establish the predictive effectiveness of variables prior to the onset of elevated blood glucose levels. An AUC of 0.79 was obtained in ML analysis conducted on the total dataset in the current study. The moderate performance can be attributed to the use of fewer variables, especially phenotypic variables instead of direct glucose or OGTT measurements, and fewer laboratory data in the current models. Moreover, the current approach employed a broader range of ML algorithms compared with previous studies, enabling a comprehensive comparison and selection of the most effective methodologies. Utilizing PyCaret for ML algorithms streamlined the process by automating data processing and model evaluation steps. Therefore, differences between this and other studies primarily stem from variations in the nature of the data.

An in-depth investigation into the predictive potential of phenotypic variables for T2DM was conducted. To complement the current ML approach, a rigorous statistical analysis was undertaken to assess the inferential strength of each individual variable. Statistical analysis tests hypotheses and infers causal relationships between individual variables and diabetes risk, while ML builds models and makes predictions based on the data, without necessarily explaining how the data are related or what causes the outcome [26]. However, statistical methods do not capture the nonlinear and interactive effects of multiple variables on diabetes risk. In contrast, ML algorithms can uncover intricate patterns and interactions within the data, which are not evident through conventional statistical measures alone. Therefore, the complementary strengths of both statistics and ML were leveraged to provide a more comprehensive understanding of the predictive factors for T2DM, allowing for the prioritization of variables that may have been overlooked by traditional statistical analysis. As Bennett et al. suggest, ML and statistical analysis are different but complementary methods that can provide different insights into the data, depending on the research question and the data available [26].

Furthermore, gender differences in diabetes risk and outcomes have been extensively reviewed in previous studies [27]. However, most of these investigations have primarily focused on the role of sex hormones, sex chromosomes, or sex-specific environmental factors in explaining these disparities. This study aimed to investigate whether phenotypic variables, including BMI, blood pressure, and lipid levels, demonstrate distinct predictive patterns for diabetes risk among males and females. By leveraging ML techniques, a substantial dataset comprising phenotypic variables was analyzed from individuals with and without T2DM. The current findings reveal that certain phenotypic variables displayed varying degrees of predictive power for diabetes risk across genders. Notably, variables like famdb, hbp, and chol exhibited higher feature importance scores for females than for males. In the SHAP analysis, the impact of heme iron intake appears more significant in males than in females when comparing Figure 6 and Figure 7. Additionally, the effect of lower activity is more pronounced in favor of diabetes in males. While higher glycemic load is more pronounced in females, it has an almost neutral effect in males. Interestingly, lower polyunsaturated fat and higher trans fat intake favor diabetes in females, but the opposite holds true for males. Furthermore, the feature importance analysis revealed gender as one of the significant factors in the overall dataset. These results suggest that phenotypic variables can capture certain facets of sex-specific pathophysiological aspects of diabetes and have the potential to enhance the accuracy and personalization of diabetes risk prediction models. Further studies are needed to validate the current findings and delve into the underlying mechanisms contributing to the sex differences observed in phenotype-based diabetes prediction.

Several recent studies have utilized PyCaret for diabetes prediction, but key differences exist between those studies and the current work. Whig et al. employed PyCaret for gestational diabetes prediction on a relatively small open-source dataset. While they reported a 90% accuracy after hyperparameter tuning, their manuscript indicated an actual accuracy of 78% [28]. This highlights the importance of dataset size, quality, and careful interpretation of results.

The study by Jose et al. shares some context with our research, but there are significant variations in datasets and outcomes. Their open-source dataset includes features beyond our scope, suggesting a focus on cardiovascular health alongside diabetes. However, it lacks crucial diabetes-related variables like family history [29]. Additionally, we leverage the more recent CatBoost algorithm from Yandex, which was absent in their work. Our study population, derived from the well-established NHS and HPFS datasets, is expected to be of higher quality.

These comparisons demonstrate key distinctions from previous research: study population, variables, algorithms, and analysis of gender differences. In previous research, authors did not mention gender as a contributing factor for the outcome, nor did they find it to contribute to predicting readmission for patients with diabetes [29]. However, our findings, supported by the statistical analysis, the feature importance plot, and the SHAP analysis, suggest that gender is a significant phenotypic factor in diabetes prediction.

## 5. Conclusions

In conclusion, this study successfully demonstrated the utility of PyCaret for T2DM prediction. However, it also highlighted the limitations of phenotypic variables in T2DM prediction due to their moderate predictive power. The combination of genotypic and phenotypic data holds promise for early T2DM detection. Previous research has demonstrated the feasibility of genotype-based T2DM prediction with high accuracy by using Logistic Regression [10]. Automated machine learning frameworks, capable of employing multiple sophisticated algorithms simultaneously, offer an avenue for novel analyses integrating genotype and phenotype data. PyCaret empowers scientists with limited coding expertise to conduct comprehensive data analysis in T2DM research, facilitating a more efficient approach by automating the entire workflow of building and deploying ML models, enabling researchers to leverage these powerful tools for diabetes research.

## Figures and Tables

**Table 1:** Variables in NHS and HPFS dataset.

Variable	Type	Description
case	Categorical	Diabetes case status; control or type 2 diabetes mellitus
famdb	Categorical	Family history of diabetes among first-degree relatives
hbp	Categorical	Reported high blood pressure
chol	Categorical	Reported high blood cholesterol at/before blood draw
bmi	Numeric	Body mass index
alcohol	Numeric	Amount of alcohol intake
heme	Numeric	Heme iron intake
magn	Numeric	Magnesium intake
ceraf	Numeric	Cereal fiber intake
pufa	Numeric	Polyunsaturated fat intake
trans	Numeric	Trans fat intake
gl	Numeric	Glycemic load
smk	Categorical	Cigarette smoking (never, past, or current)
act	Numeric	Exercise habits (total physical activity)
Gender	Categorical	Male/female

**Table 2:** Age and numerical phenotypic characteristics of control and T2DM individuals.

	Male			Female		
	Control	T2DM	*p*-Value	Control	T2DM	*p*-Value
n	1289	1127		1774	1482	
Age (year)	59.22 ± 8.36	59.38 ± 8.48	0.64	55.63 ± 6.74	55.97 ± 6.76	0.16
Body mass index (kg/m^2^)	25.21 ± 2.83	27.89 ± 4.13	1.69 × 10^−73^	25.36 ± 4.80	29.88 ± 5.73	2.75 × 10^−121^
Activity (MET hours/week)	40.8 ± 42.96	29.98 ± 33.10	5.77 × 10^−15^	15.57 ± 18.50	12.86 ± 15.61	3.69 × 10^−8^
Alcohol (Gram/day)	11.05 ± 15.02	9.57 ± 14.69	2.26 × 10^−5^	6.55 ± 10.32	4.04 ± 9.44	4.38 × 10^−11^
Polyunsaturated fatty acid (Energy%)	5.88 ± 1.55	6.12 ± 1.68	1.1 × 10^−4^	6.32 ± 1.68	6.17 ± 1.57	0.044
Trans (Energy%)	1.51 ± 0.62	1.56 ± 0.61	0.023	1.69 ± 0.53	1.73 ± 0.55	0.065
Magnesium (mg/day)	386.01 ± 87.67	378.08 ± 86	0.029	304.66 ± 71.83	300.29 ± 69.12	0.068
Ceraf (Gram/day)	7.26 ± 4.33	6.61 ± 3.8	7.22 × 10^−05^	4.59 ± 3.19	4.39 ± 2.74	0.085
Heme (mg/day)	1.19 ± 0.45	1.34 ± 0.5	4.44 × 10^−16^	1.13 ± 0.45	1.2 ± 0.45	1.71 × 10^−5^
Glycemic load	130.85 ± 25.75	124.14 ± 24.38	4.88 × 10^−11^	97.52 ± 19.58	98.58 ± 18.5	0.052

Data are presented as means ± standard deviation. n denotes number of individuals; T2DM: type 2 diabetes mellitus; and MET: metabolic equivalents. The *p*-value represents the statistical significance of the difference between male and female sample means in the Student’s *t*-test analysis for body mass index and the Mann–Whitney U test for other variables. The glycemic load is a unitless measure. It reflects the equivalent effect on blood sugar of consuming a particular food compared with ingesting one gram of pure glucose.

**Table 3:** The table shows the distribution of categorical data, including family history of diabetes (famdb), high blood pressure (hbp), cholesterol, and cigarette smoking, across gender and disease status.

		Male						Female					
		Control		T2DM		χ^2^	*p*-Value	Control		T2DM		χ^2^	*p*-Value
		n	%	n	%			n	%	n	%		
Famdb	no	1014	78.7	642	57.0	131.32	<0.00001	1382	77.9	749	50.5	267.35	<0.00001
	yes	275	21.3	485	43.0			392	22.1	733	49.5		
Hbp	no	1008	78.2	667	59.2	102.26	<0.00001	1421	80.1	760	51.3	303.24	<0.00001
	yes	281	21.8	460	40.8			353	19.9	722	48.7		
Cholesterol	no	923	71.7	668	59.3	40.67	<0.00001	1585	89.3	1134	76.5	96.47	<0.00001
	yes	366	28.3	459	40.7			189	10.7	348	23.5		
Cigarette smoking	current	75	6.0	102	9.3	26.70	<0.00001	190	10.8	210	14.2	14.97	<0.00056
	never	597	47.8	415	38.0			883	50.0	652	44.0		
	past	576	46.2	576	52.7			693	39.2	618	41.8		

The data are presented as counts and percentages. χ^2^: chi-square test value.

**Table 4:** Performance of 16 machine learning techniques for predicting diabetes in male-only data subset.

	Model	Accuracy	AUC	Recall	Prec.	F1	Kappa	MCC	TT (s)
**ridge**	Ridge Classifier	0.7013	0.0000	0.7013	0.7024	0.6991	0.3956	0.3989	0.0360
**lda**	Linear Discriminant Analysis	0.7013	0.7676	0.7013	0.7024	0.6991	0.3956	0.3989	0.0350
**lr**	Logistic Regression	0.7002	0.7670	0.7002	0.7007	0.6983	0.3940	0.3963	0.6500
**gbc**	Gradient Boosting Classifier	0.6919	0.7447	0.6919	0.6930	0.6900	0.3778	0.3805	0.2010
**nb**	Naive Bayes	0.6877	0.7406	0.6877	0.6880	0.6857	0.3688	0.3710	0.0320
**ada**	Ada Boost Classifier	0.6865	0.7397	0.6865	0.6868	0.6846	0.3667	0.3687	0.0810
**catboost**	CatBoost Classifier	0.6801	0.7435	0.6801	0.6804	0.6784	0.3540	0.3559	1.3040
**rf**	Random Forest Classifier	0.6771	0.7339	0.6771	0.6776	0.6759	0.3496	0.3509	0.1680
**et**	Extra Trees Classifier	0.6759	0.7349	0.6759	0.6759	0.6754	0.3479	0.3484	0.1190
**xgboost**	Extreme Gradient Boosting	0.6694	0.7129	0.6694	0.6700	0.6683	0.3340	0.3355	0.1090
**lightgbm**	Light Gradient Boosting Machine	0.6635	0.7124	0.6635	0.6635	0.6622	0.3214	0.3227	0.0670
**svm**	SVM—Linear Kernel	0.5973	0.0000	0.5973	0.6642	0.5422	0.1845	0.2290	0.0380
**dt**	Decision Tree Classifier	0.5949	0.5932	0.5949	0.5960	0.5938	0.1865	0.1874	0.0370
**qda**	Quadratic Discriminant Analysis	0.5878	0.6125	0.5878	0.5950	0.5821	0.1772	0.1816	0.0390
**dummy**	Dummy Classifier	0.5334	0.5000	0.5334	0.2845	0.3711	0.0000	0.0000	0.0460
**knn**	K Neighbors Classifier	0.5204	0.5242	0.5204	0.5173	0.5167	0.0298	0.0300	0.0390

**Table 5:** Performance of 16 machine learning techniques for predicting diabetes in female-only data subset.

	Model	Accuracy	AUC	Recall	Prec.	F1	Kappa	MCC	TT (s)
**lr**	Logistic Regression	0.7121	0.7891	0.7121	0.7126	0.7107	0.4161	0.4182	0.8070
**ridge**	Ridge Classifier	0.7099	0.0000	0.7099	0.7104	0.7078	0.4103	0.4132	0.0890
**lda**	Linear Discriminant Analysis	0.7099	0.7897	0.7099	0.7104	0.7078	0.4103	0.4132	0.0970
**ada**	Ada Boost Classifier	0.7056	0.7700	0.7056	0.7061	0.7045	0.4043	0.4059	0.2810
**catboost**	CatBoost Classifier	0.7003	0.7748	0.7003	0.7020	0.7003	0.3972	0.3981	3.2050
**nb**	Naive Bayes	0.6985	0.7601	0.6985	0.6997	0.6983	0.3924	0.3934	0.0950
**rf**	Random Forest Classifier	0.6977	0.7639	0.6977	0.6998	0.6972	0.3913	0.3930	0.5160
**gbc**	Gradient Boosting Classifier	0.6950	0.7741	0.6950	0.6971	0.6948	0.3867	0.3880	0.6540
**et**	Extra Trees Classifier	0.6867	0.7599	0.6867	0.6872	0.6862	0.3677	0.3685	0.3680
**lightgbm**	Light Gradient Boosting Machine	0.6832	0.7479	0.6832	0.6842	0.6830	0.3618	0.3625	0.1750
**xgboost**	Extreme Gradient Boosting	0.6713	0.7351	0.6713	0.6719	0.6709	0.3369	0.3376	0.3380
**qda**	Quadratic Discriminant Analysis	0.6371	0.6722	0.6371	0.6462	0.6366	0.2776	0.2820	0.0910
**dt**	Decision Tree Classifier	0.6218	0.6177	0.6218	0.6218	0.6206	0.2355	0.2365	0.1170
**svm**	SVM—Linear Kernel	0.5994	0.0000	0.5994	0.6714	0.5285	0.1799	0.2210	0.0980
**knn**	K Neighbors Classifier	0.5884	0.6089	0.5884	0.5862	0.5858	0.1638	0.1648	0.1120
**dummy**	Dummy Classifier	0.5450	0.5000	0.5450	0.2970	0.3845	0.0000	0.0000	0.0930

**Table 6:** Performance of 16 ML techniques for predicting diabetes in total (male + female) dataset.

	Model	Accuracy	AUC	Recall	Prec.	F1	Kappa	MCC	TT (s)
**lr**	Logistic Regression	0.7174	0.7914	0.7174	0.7172	0.7160	0.4276	0.4291	0.7600
**gbc**	Gradient Boosting Classifier	0.7169	0.7847	0.7169	0.7173	0.7167	0.4301	0.4305	0.3990
**catboost**	CatBoost Classifier	0.7161	0.7818	0.7161	0.7164	0.7157	0.4277	0.4284	1.7910
**ada**	Ada Boost Classifier	0.7149	0.7807	0.7149	0.7146	0.7138	0.4233	0.4244	0.1400
**lda**	Linear Discriminant Analysis	0.7146	0.7912	0.7146	0.7145	0.7129	0.4213	0.4233	0.0440
**ridge**	Ridge Classifier	0.7144	0.0000	0.7144	0.7143	0.7126	0.4207	0.4228	0.0440
**nb**	Naive Bayes	0.7103	0.7660	0.7103	0.7102	0.7101	0.4163	0.4165	0.0450
**lightgbm**	Light Gradient Boosting Machine	0.7073	0.7764	0.7073	0.7075	0.7070	0.4101	0.4107	0.0900
**rf**	Random Forest Classifier	0.7058	0.7763	0.7058	0.7066	0.7056	0.4080	0.4087	0.3510
**et**	Extra Trees Classifier	0.7008	0.7664	0.7008	0.7015	0.7007	0.3981	0.3986	0.2010
**xgboost**	Extreme Gradient Boosting	0.6965	0.7554	0.6965	0.6968	0.6963	0.3888	0.3892	0.2040
**qda**	Quadratic Discriminant Analysis	0.6710	0.7191	0.6710	0.6740	0.6657	0.3307	0.3371	0.0500
**dt**	Decision Tree Classifier	0.6262	0.6243	0.6262	0.6268	0.6263	0.2484	0.2486	0.0600
**svm**	SVM—Linear Kernel	0.6065	0.0000	0.6065	0.6409	0.5384	0.1926	0.2436	0.0550
**dummy**	Dummy Classifier	0.5401	0.5000	0.5401	0.2917	0.3788	0.0000	0.0000	0.0500

## Data Availability

phs000091 samples are available at https://www.ncbi.nlm.nih.gov/gap database, (accession number: phs000091.v2.p1, accessed on 1 June 2024).
